# Detecting N-myristoylation and S-acylation of host and pathogen proteins in plants using click chemistry

**DOI:** 10.1186/s13007-016-0138-2

**Published:** 2016-08-03

**Authors:** Patrick C. Boyle, Simon Schwizer, Sarah R. Hind, Christine M. Kraus, Susana De la Torre Diaz, Bin He, Gregory B. Martin

**Affiliations:** 1 Boyce Thompson Institute for Plant Research, Ithaca, NY 14853 USA; 2 Plant Pathology and Plant–Microbe Biology Section, School of Integrative Plant Science, Cornell University, Ithaca, NY 14853 USA; 3 Department of Chemistry and Chemical Biology, Cornell University, Ithaca, NY 14853 USA; 4 Monsanto Company, St. Louis, MO 63141 USA; 5 College of Pharmacy, Guiyang Medical University, Guiyang, 550004 Guizhou China

**Keywords:** Fatty acylation, Myristoylation, Palmitoylation, Stearylation, S-acylation, Click chemistry, Plasma membrane, Pathogen effectors, Pattern recognition receptors, Resistance proteins, *Arabidopsis thaliana*, *Nicotiana benthamiana*

## Abstract

**Background:**

The plant plasma membrane is a key battleground in the war between plants and their pathogens. Plants detect the presence of pathogens at the plasma membrane using sensor proteins, many of which are targeted to this lipophilic locale by way of fatty acid modifications. Pathogens secrete effector proteins into the plant cell to suppress the plant’s defense mechanisms. These effectors are able to access and interfere with the surveillance machinery at the plant plasma membrane by hijacking the host’s fatty acylation apparatus. Despite the important involvement of protein fatty acylation in both plant immunity and pathogen virulence mechanisms, relatively little is known about the role of this modification during plant-pathogen interactions. This dearth in our understanding is due largely to the lack of methods to monitor protein fatty acid modifications in the plant cell.

**Results:**

We describe a rapid method to detect two major forms of fatty acylation, N-myristoylation and S-acylation, of candidate proteins using alkyne fatty acid analogs coupled with click chemistry. We applied our approach to confirm and decisively demonstrate that the archetypal pattern recognition receptor FLS2, the well-characterized pathogen effector AvrPto, and one of the best-studied intracellular resistance proteins, Pto, all undergo plant-mediated fatty acylation. In addition to providing a means to readily determine fatty acylation, particularly myristoylation, of candidate proteins, this method is amenable to a variety of expression systems. We demonstrate this using both Arabidopsis protoplasts and stable transgenic Arabidopsis plants and we leverage Agrobacterium-mediated transient expression in *Nicotiana benthamiana* leaves as a means for high-throughput evaluation of candidate proteins.

**Conclusions:**

Protein fatty acylation is a targeting tactic employed by both plants and their pathogens. The metabolic labeling approach leveraging alkyne fatty acid analogs and click chemistry described here has the potential to provide mechanistic details of the molecular tactics used at the host plasma membrane in the battle between plants and pathogens.

**Electronic supplementary material:**

The online version of this article (doi:10.1186/s13007-016-0138-2) contains supplementary material, which is available to authorized users.

## Background

The covalent attachment of fatty acids to specific protein residues, a process referred to as fatty acylation, increases the hydrophobicity of the substrate protein and affects various properties, most notably subcellular localization [1, 2]. These lipid moieties often serve as hydrophobic anchors that promote protein-membrane associations [1]. There are a number of different types of protein fatty acylations, the two best characterized forms in plants being N-myristoylation and S-acylation [2].

N-myristoylation describes the irreversible amide bond formation between myristate, a saturated 14-carbon fatty acid, and the N-terminal amine of a glycine residue exposed as a result of co-translational N-terminal methionine excision, or more rarely, post-translational proteolytic processing [1, 3, 4]. This modification is mediated by N-myristoyltransferases, cytosolic entities often associated with ribosomes since protein myristoylation is typically a co-translational modification [5–8]. In many cases, myristoylation is necessary for targeting a protein to the plasma membrane (PM), but this modification alone is not sufficient to provide permanent anchoring to the membrane and as a result myristoylation is often found in combination with other membrane interaction motifs, including polybasic domains and those involving S-acylation [1, 3, 4].

S-acylation refers to the reversible thioester bond formation between a fatty acid and a cysteine residue side chain [9]. The saturated 16-carbon palmitate is the fatty acid most frequently featured in S-acylation and therefore this modification is often termed palmitoylation [1, 10]. However, other fatty acids can be covalently attached to cysteine side chains, most notably the saturated 18-carbon stearate, and a small number of studies suggest that in plants, protein stearylation is as prevalent as palmitoylation [2, 11–13]. The enzymes responsible for S-acylation are known as S-acyltransferases, or more commonly, palmitoyl acyltransferases [14]. These enzymes are integral membrane proteins found at the PM and at the membranes of various cellular compartments, including endosomes, the Golgi apparatus, and the endoplasmic reticulum [14]. Unlike myristoylation, protein S-acylation with palmitate or stearate is sufficient for stable interaction with the membrane [2, 15]. This modification is suggested to serve roles in retaining proteins at various membranes and trafficking previously myristoylated proteins to the PM, in addition to dynamically regulating protein activity, stability, and complex assembly [1, 9, 14]. Proteins bearing both myristoylation and proximal S-acylation are said to be N-terminally dual fatty acylated and this combination of lipid modifications appears to drive stable association with the PM [1].

Fatty acylation is a form of protein modification that is conserved among eukaryotes and most of the information available about this modification is based on studies from yeast and animal systems. However, what little is known about myristoylation and S-acylation in plants suggests that this kingdom is sufficiently unique in its use of these modifications to merit independent investigation [6, 16–20]. Prediction based studies indicate that the plant proteome is proportionally more myristoylated than those of metazoans and fungi [6, 16, 17, 19]. Interestingly, many of the protein families predicted to be myristoylated exclusively in plants are implicated in stress and defense responses [6, 19].

In contrast to the absolute requirement of an N-terminal glycine for myristoylation, S-acylation does not have a clear consensus sequence beyond a requisite cysteine residue, which can occur at essentially any position in a protein [20]. The lack of an S-acylation consensus sequence has largely prevented the use of predictive bioinformatics approaches to study this modification [10, 21, 22]. However, the labile nature of thioester bonds has permitted the use of an acyl-biotin exchange (ABE) approach to identify S-acylated proteins present in the plant proteome. A recent study based on the ABE method indicated that more than 500 proteins are subject to S-acylation in Arabidopsis root suspension cells, which far exceeds the number of Arabidopsis proteins predicted to be myristoylated [20]. Similar to what has been reported with plant proteins subject to myristoylation, many of the proteins identified as being S-acylated appear to be involved in pathogen perception [20]. The prevalence for fatty acylation of proteins functioning in defense is not unexpected because these modifications are known to target proteins to the PM and this lipophilic locale constitutes the initial point of pathogen perception in plants [2]. The organization of the plant palmitoyl acyltransferases, which are present at the PM in greater proportions than in mammalian and yeast systems, suggests that the plant S-acylation apparatus is uniquely arranged for the stable recruitment of proteins to this particular membrane locale [14].

The use of host-mediated myristoylation by plant pathogen effectors supports suggestions that the plant PM is a critical interface during plant-pathogen interactions and that the fatty acylation mechanisms are distinctively organized and/or accessible in the plant cell environment [23]. The exploitation of host-mediated protein lipidation mechanisms for the spatial regulation of pathogen effectors seems to be a general virulence strategy, yet only the effectors of plant pathogenic bacteria appear to hijack the host myristoylation machinery [24–31]. To date, the reason for this observation remains unclear, but the modification is essential for the virulence activity of several bacterial effectors and is required for the recognition of many effectors in host plants armed with the appropriate intracellular sensor proteins, more commonly referred to as resistance proteins [24, 32–34].

Despite the distinct features of protein fatty acylation in plants and its importance in plant-pathogen interactions, methods to readily and decisively detect specific fatty acid modifications of host and pathogen proteins in the plant cell are currently lacking. The ability to monitor plant-mediated myristoylation has proven particularly problematic because of the irreversible nature of this modification. Traditional approaches to directly demonstrate protein fatty acylation in vivo have relied on metabolic labeling with radiolabeled fatty acids, such as [^3^H]- or [^125^I]-myristic and palmitic acids, followed by purification of the protein of interest and visualization using autoradiography [35]. This method, although effective, typically requires lengthy film exposure times to visualize fatty acylated proteins and requires the use of radioactive materials [3, 4, 34, 35]. Furthermore, radiolabeling techniques do not present any straightforward means to capture labeled proteins, preventing proteome-wide identification of fatty acylated targets [35]. Lipid modification analysis by gas chromatography coupled with mass spectrometry (GC–MS) is another approach that has advanced our understanding of candidate protein S-acylation, particularly in plants [36]. The advantages of this approach are its ability to unambiguously identify S-acylation modifications, such as palmitoylation and stearylation, and to do so without the requirement of feeding radiolabeled materials to the cells or tissues being interrogated [37]. However this technique cannot be applied to the analysis of protein myristoylation and, like radiolabeling approaches, is not amenable to whole proteome analysis. The ABE approach was developed as a relatively rapid nonradioactive alternative to study protein fatty acylation and enables proteome-wide identification of S-acylated proteins [38, 39]. ABE leverages the labile nature of thioester linkages to replace S-acylation modifications present on cysteine residues with a chosen label, most often biotin. The labeled proteins are then enriched on affinity resin and subsequently identified using mass spectrometry. Alternatively, the labeled proteins can be visualized by in-gel fluorescence or western blotting. However, the ABE method has some limitations, most notably that this approach, like the GC–MS strategy, can only be applied to study S-acylation and not myristoylation [35]. Also, due to its indirect nature the technique does not allow for the discernment of different thioester linkages, many of which are not involved in S-acylation, and therefore results in false positives [20, 35, 39–41].

Metabolic labeling approaches using fatty acid analogs containing bio-orthogonal chemical handles, which allow for the attachment of reporter or detection tags, have recently emerged as means to circumvent many of the difficulties that have impeded the study of fatty acid modifications [35, 42–45]. The strategy involves feeding cells a fatty acid analog bearing a bio-orthogonal azide or alkyne handle, resulting in metabolic incorporation of the analog into target proteins. Click chemistry is then used to react the bio-orthogonal functionality present in the fatty acid analog with a reporter tag. These fatty acids are termed bio-orthogonal analogs because the sleek nature of the azide or alkyne handles present in terminal positions of these modified lipids interfere neither with the hydrophobic character of these molecules nor the acid moiety, preserving the ability to insert into membranes and interact with the native fatty acylation apparatus which allows their metabolic incorporation into target proteins [45]. These chemical tools enable the rapid detection of protein myristoylation and S-acylation without the need for radioactivity [35]. Click chemistry, based primarily on the Huisgen [3 + 2] Cu(I)-catalyzed azide-alkyne cycloaddition, has an ever growing number of applications and has been employed in plant systems to determine the targets of reactive small molecules, visualize cell lignification, track Golgi protein dynamics, and detect protein prenylation, but it has not yet been leveraged to study fatty acid modifications of plant proteins [46–49].

Protein fatty acylation in plants has many interesting features compared to other eukaryotes. However, many questions still remain about the role of these protein modifications in this kingdom due to the lack of techniques currently available to study fatty acylation, particularly post-translational myristoylation, in plants. Here we describe the development of a click chemistry-based method using ω-alkynyl fatty acid analogs to facilitate the study of fatty acylation of both host and pathogen effector proteins in the plant cell environment.

## Results

### General scheme for assessing fatty acylation of candidate proteins using clickable fatty acid analogs

**Fig. 1:**
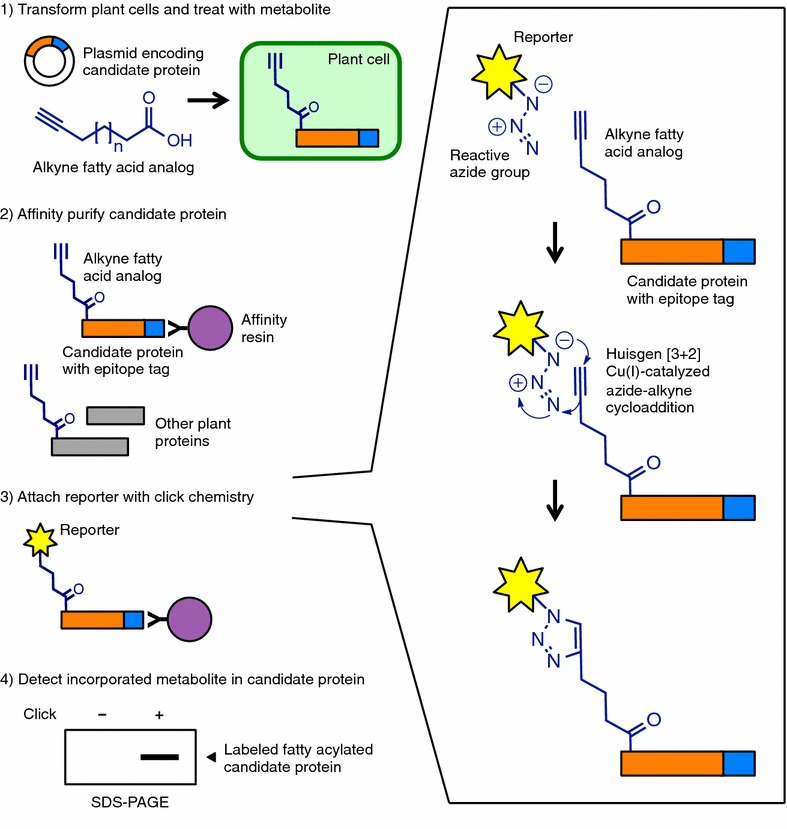
Experimental scheme for assessing fatty acylation of proteins in plant cells using clickable fatty acid analogs. Adapted from [46]

We developed and optimized an approach to determine the fatty acylation status, especially myristoylation, of candidate proteins in plant cells using ω-alkynyl fatty acid analogs and click chemistry based largely on methods previously described [45, 50] (Fig. 1). Plant cells are transformed with a candidate gene construct, preferably encoding a commercial epitope tag, following standard protocols. The alkyne fatty acid analog for the metabolite of interest is applied to the plant cells and subsequently incorporated during protein synthesis. Total protein is extracted and the candidate protein purified using immunoprecipitation. A reporter, such as a biotin tag or a fluorescent dye, is added to the alkyne group of the fatty acid analog using click chemistry and detected by western blotting or fluorescence imaging (Fig. 1). The experimental steps outlined here can be completed within a few days and we describe below the successful application of this approach to detect fatty acid modifications in a variety of candidate proteins using different expression methods and plant systems.

### Alkyne fatty acid analogs are better tolerated by plant cells than preparations of azide fatty acid analogs

To assess the potential phytotoxicity of different forms of fatty acid analogs, we transformed Arabidopsis protoplasts with an expression vector encoding yellow fluorescent protein (YFP) and treated the protoplasts with either Az12 or Alk12, which are the azide- and alkyne-functionalized myristic acid analogs, respectively (Additional file 1: Figure S1). We found that even very low concentrations of Az12 strongly diminished YFP accumulation, whereas Alk12 showed inhibitory effects only at relatively high concentrations (Additional file 1: Figure S1). Therefore, we decided to perform all subsequent experiments using preparations of alkyne fatty acid analogs.

### The pattern recognition receptor FLS2 is S-acylated

The plant PM is armed with a series of sensors that function as a surveillance system to detect the presence of invading microbes and much of the machinery involved in monitoring this crucial lipophilic locale features some form of fatty acylation [23, 51]. These PM-localized pattern recognition receptors (PRRs) are able to perceive the presence of pathogens through the recognition of conserved microbe-associated molecular patterns [51]. Activation of PRRs stimulates signaling through intracellular protein kinases, resulting in the deployment of a broad-spectrum defense response referred to as pattern-triggered immunity (PTI) [52].

**Fig. 2:**
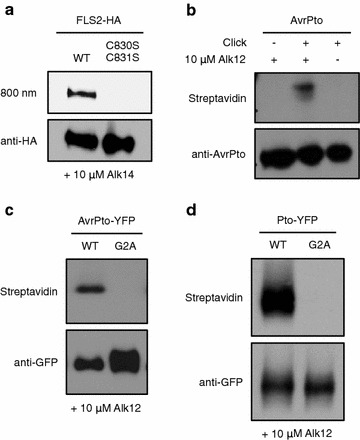
Fatty acid modifications of proteins involved in plant immunity. **a** Arabidopsis protoplasts were transformed with HA epitope-tagged *FLS2* wild-type (WT) or an *fls2* mutant encoding C830S, C831S. Protoplasts were treated with 10 μM Alk14, incubated for 6 h, and cells collected. Total protein was extracted, FLS2 proteins immunoprecipitated using anti-HA resin, and click chemistry performed. Incorporated Alk14 was visualized by fluorescence imaging and total protein was detected by anti-HA western blotting. **b** Transgenic Arabidopsis plants conditionally expressing *avrPto* were treated with 20 μM dexamethasone to induce transgene expression. Leaves were infiltrated twice with 10 μM Alk12, 6 h after induction and 6 h before sampling. Tissue was collected 30 h after induction and total protein extracted. AvrPto was immunoprecipitated using anti-AvrPto resin and a biotin tag added using click chemistry. Streptavidin-HRP western blotting was used to detect incorporation of Alk12. Anti-AvrPto western blotting was used to verify equal amounts of protein in all samples. **c** *Nicotiana benthamiana* leaves were infiltrated with Agrobacterium strains carrying *avrPto*-*YFP* fusion constructs encoding the WT protein or a G2A mutant. 10 μM Alk12 was infiltrated twice, 24 h after Agrobacterium infiltration and 6 h before sampling. Tissue was collected 48 h after transformation and total protein extracted. AvrPto proteins were immunoprecipitated using anti-GFP resin and a biotin tag attached using click chemistry. Incorporated Alk12 was detected by streptavidin-HRP western blotting. The anti-GFP western blot shows relative protein levels. **d** *Nicotiana benthamiana* was used to transiently express *Pto*-*YFP* fusions encoding the WT protein or a G2A mutant. 10 μM Alk12 was infiltrated twice, 24 h after Agrobacterium infiltration and 6 h before sampling. Tissue was collected 48 h after transformation, total protein extracted, and Pto proteins immunoprecipitated using anti-GFP resin. A biotin tag was attached using click chemistry and incorporation of Alk12 was detected by streptavidin-HRP western blotting. Protein levels were visualized by anti-GFP western blotting

One of the best-characterized PRRs is Arabidopsis flagellin-sensitive 2 (FLS2), which recognizes a highly conserved 22-amino acid sequence from the N-terminal portion of bacterial flagellin [53, 54]. A recent survey of protein S-acylation in Arabidopsis using an ABE approach strongly suggested that FLS2 is S-acylated at cysteine residues 830 and 831 [55, 56]. To validate our click chemistry-based approach and to directly show incorporation of palmitic acid at these sites, we expressed wild-type *FLS2* and a mutant encoding serine substitutions at residues 830 and 831 (C830S, C831S) in Arabidopsis protoplasts in the presence of the palmitic acid analog Alk14 (Fig. 2a). Following protein extraction and immunoprecipitation, we introduced a fluorescent reporter tag using click chemistry to visualize Alk14 incorporation. We were able to detect a strong fluorescent signal only with wild-type FLS2, and not the C830S, C831S mutant. Anti-HA western blotting showed comparable accumulation of the two proteins (Fig. 2a). Importantly, given that the C830S, C831S mutant is targeted to the PM like wild-type FLS2 [20], the lack of labeling observed with the mutant indicates that fatty acid analogs are not attached to these proteins simply due to their proximity to the lipid-rich PM. Taken together, this result demonstrates that our approach is well suited to study the fatty acylation status of candidate proteins in plant cells.

### Alkyne fatty acid analogs do not appear to interfere with programmed cell death and permeate intact cells in leaf tissue

The alkyne-functionalized fatty acid analogs do not appear to interfere with protein synthesis when used at moderate concentrations and are readily incorporated into proteins in a protoplast system. However, it remained possible that in the context of whole leaf tissue these analogs could cause spurious cell death symptoms, interfere with certain immune responses, or are unable to permeate intact cells. To address these concerns, we tested if the tomato resistance protein Pto, which mediates recognition of the *Pseudomonas syringae* pv. *tomato* effector AvrPto, retains the ability to trigger programmed cell death (PCD) in the presence of the different alkyne fatty acid analogs [57–59]. We transiently expressed *Pto* together with *avrPto* or an empty vector in *Nicotiana benthamiana* leaf tissue using Agrobacterium-mediated transformation, followed by infiltration of the alkyne fatty acid analogs (Additional file 2: Figure S2A). We found that neither the myristic acid analog Alk12, the palmitic acid analog Alk14, nor the stearic acid analog Alk16 produced any spurious symptoms nor did they affect PCD in response to AvrPto, even though all of the alkyne-bearing metabolites were used at high concentrations (Additional file 2: Figure S2A), suggesting that they are suitable to study the role of protein fatty acylation in plant-pathogen interactions.

To ensure that the metabolites are able to permeate intact plant cells and label fatty acylated proteins in the context of whole leaf tissue, we transiently expressed the *avrPto* effector in *N. benthamiana* leaves and syringe-infiltrated preparations of the alkyne fatty acid analogs into the transformed leaf tissue [24, 60] (Additional file 2: Figure S2B). We chose this particular protein to test the ability of the three fatty acid analogs for cell permeation and protein incorporation because AvrPto contains a predicted dual fatty acylation motif suggesting that it is subject to both plant-mediated myristoylation and S-acylation [32, 61]. We performed whole protein extraction, affinity purified the epitope-tagged AvrPto, and attached a fluorescent dye using click chemistry as described for FLS2. We visualized incorporation of the different alkyne fatty acid analogs using fluorescence imaging and detected incorporation of all three probes, although with varying signal strength (Additional file 2: Figure S2A). This demonstrates that the alkyne fatty acid analogs are able to permeate intact leaf cells and are likely incorporated by way of innate metabolic processes, making this approach suitable for the study of protein fatty acylation in leaf tissue.

### A transgenic Arabidopsis line shows incorporation of a myristic acid analog into AvrPto

Bacterial plant pathogens employ the type III secretion system to inject effector proteins directly into the plant cell to subvert PTI signaling, ultimately rendering the host susceptible to infection [52, 62]. Spatial regulation of effectors is required for their virulence function because it ensures that they engage their intended targets and enhances the local concentration of these pathogen-derived proteins, which are likely delivered into the host cell in very small amounts [30]. Several effectors have been shown to target the PTI machinery present at the intracellular face of the plant PM and a number of these bacterial proteins appear to hijack the host fatty acylation apparatus to access this lipophilic locale [24, 32, 34, 63]. Notably, plant-mediated fatty acylation has not been decisively demonstrated for most effectors, but rather inferred from studies showing that N-terminal glycine and/or cysteine substitutions prevent PM localization and render the effectors unable to exert their virulence function or elicit an immune response, depending on the host plant [32–34]. A recent review elaborates on the exploitation of host-mediated fatty acylation by plant pathogenic effectors [23].

The AvrPto effector promotes bacterial pathogenesis by targeting the FLS2 receptor complex in order to suppress flagellin perception [64, 65]. Like the PM-associated FLS2, AvrPto was shown to localize to the cell periphery [32, 66, 67]. It is strongly suggested that targeting of this pathogen protein to the plant PM requires post-translational host-mediated myristoylation of the glycine-2 (G2) residue in AvrPto, since the G2A mutation abolishes both PM localization and virulence function of the effector [32, 66]. To test if AvrPto is indeed myristoylated in plant cells, we took advantage of a transgenic Arabidopsis line conditionally expressing *avrPto* under control of a dexamethasone-inducible system [68] (Fig. 2b). We syringe-infiltrated leaves with the myristic acid analog Alk12 following dexamethasone treatment, extracted total protein, and immunoprecipitated AvrPto using anti-AvrPto resin. The incorporated fatty acid was then biotinylated using click chemistry and visualized by western blotting. We were able to detect a specific band of the expected size in samples treated with Alk12 and subsequently subjected to click chemistry. To control for unspecific detection in the absence of an AvrPto G2A mutant, we included samples without performing click chemistry and without infiltrating the metabolite. Anti-AvrPto western blotting was used to verify equal amounts of the effector protein in all samples (Fig. 2b). This result shows that AvrPto is myristoylated in plant cells and demonstrates that our approach can be applied to assess fatty acylation of candidate proteins stably expressed in transgenic Arabidopsis leaf tissue. However, the lack of a stable Arabidopsis line expressing a G2 point mutant of AvrPto prevented us from determining whether this modification is mediated by way of canonical G2 myristoylation or not; we address this limitation in the next section.

### Transient expression in *N. benthamiana* demonstrates that AvrPto is myristoylated at its N-terminus

To establish a higher-throughput system for validation of multiple candidate proteins, we took advantage of Agrobacterium-mediated transient gene expression in *N. benthamiana*. This approach also enables the use of point mutants to validate predicted fatty acylation sites and to control for nonspecific incorporation of alkyne fatty acid analogs. We transiently expressed *avrPto* variants and infiltrated the leaf tissue with the myristic acid analog Alk12 (Fig. 2c). To counter previously observed instability of the AvrPto G2A mutant, we fused the effector to YFP in an attempt to stabilize the protein. We were able to detect click-mediated biotinylation using streptavidin with wild-type AvrPto, but no band was detected with the predicted myristoylation mutant G2A despite high protein accumulation (Fig. 2c). Thus, using the *N. benthamiana* system, we were able to extend our data obtained in Arabidopsis to conclusively show typical G2-mediated myristoylation of AvrPto through the use of a specific point mutant.

As previously mentioned, the AvrPto N-terminus contains a predicted dual fatty acylation motif, MGNICVGGSR, due to the G2 and proximal cysteine-5 (C5) residues [32, 61]. While we showed labeling of this pathogen effector with Alk12, Alk14, and Alk16 (Additional file 2: Figure S2B), we were unable to map S-acylation type modifications, by Alk14 or Alk16, to the C5 position due to instability and inconsistent labeling of the AvrPto mutant forms (data not shown). However, it is likely that AvrPto is S-acylated at the C5 position because this residue is the only cysteine present in the effector protein.

### Detection of Pto myristoylation in plant cells is greatly enhanced using metabolic labeling coupled with click chemistry

Plants have evolved intracellular surveillance mechanisms to perceive the presence and activity of pathogen effectors [69]. Detection of effectors within the host cell indicates infection by an adapted pathogen and as a result the plant activates an amplified defense response referred to as effector-trigger immunity (ETI) which is often associated with PCD [69, 70]. ETI signaling is typically mediated by nucleotide-binding leucine-rich repeat (NB-LRR) proteins that are often physically partnered with either a decoy which resembles a host protein targeted by effectors, or an actual host target [71, 72]. In either case, interactions between effectors and host proteins are sensed by the associated NB-LRRs which subsequently activate ETI [72]. Regardless of the specific mode of detection, the precise localization of these surveillance mechanisms is critical to their function and because the intracellular face of the plant PM is an area intensely attacked by effectors, many of these sensors are positioned at this crucial locale by way of lipid modifications [72–77].

Some tomato accessions rely upon the Pto kinase, acting in concert with the NB-LRR protein Prf, to recognize the fatty acylated pathogen effector AvrPto [78–82]. Pto appears to function as a decoy that mimics the structure of the kinase domains present in PRR signaling complexes, such as that of FLS2, but in contrast to the PM-spanning receptors it is proposed to mimic, Pto lacks a transmembrane domain [78]. Previous work using a radiolabeled myristic acid feeding approach showed that Pto is myristoylated in plant cells and that the G2 residue associated with myristoylation is required for full recognition of AvrPto [60, 83]. To confirm incorporation of myristic acid with our click chemistry-based approach, we transiently expressed wild-type *Pto* and a mutant encoding the G2A substitution in *N. benthamiana* (Fig. 2d). Following the strategy used for AvrPto, we fused Pto to YFP to stabilize the G2A mutant as demonstrated by the anti-GFP western blot. We were able to detect incorporation of the myristic acid analog Alk12 into wild-type Pto using streptavidin after adding a biotin tag via click chemistry. No band was detected for the G2A mutant (Fig. 2d). This experiment confirms Pto myristoylation and extends our click chemistry-based method to assess fatty acylation to include a PM receptor, a pathogen effector, and an intracellular host resistance protein.

### Myristoylome labeling using alkyne fatty acid analogs

The strict requirement of an N-terminal glycine residue coupled with the availability of plant genome sequences has enabled the prediction of myristoylated proteins across the proteome, a collection of proteins also referred to as the myristoylome [6, 16, 17, 19]. However, methods to directly validate the predicted myristoylome in plant cells are lacking. Furthermore, bioinformatic approaches are unable to predict non-canonical myristoylation, such as the post-translational protein myristic acid modification required for the virulence function and recognition of the bacterial effector AvrPphB in plant cells [34]. To begin to address these limitations, our click chemistry-based approach could be modified and applied to enrich and investigate the myristoylome and potentially enable proteome-wide analysis of other fatty acid modifications in plants [6, 16, 17]. We performed a pilot experiment using AvrPto and transiently expressed the effector in *N. benthamiana* leaf tissue and subsequently introduced the myristic acid analog Alk12 by infiltration (Additional file 3: Figure S3). Total protein was extracted, a standard methanol/chloroform precipitation performed, and click chemistry used to biotinylate the incorporated Alk12. A second methanol/chloroform precipitation was used to remove unreacted azido-biotin prior to affinity purification using streptavidin resin. We interrogated this biotinylated material for the presence of AvrPto by anti-HA western blotting and were able to detect the effector from among the multitude of biotinylated proteins (Additional file 3: Figure S3). This result demonstrates that using the described protocol it is possible to capture a myristoylated protein from among a complex plant lysate by way of its fatty acid modification. Admittedly, the AvrPto protein used in this pilot experiment was overexpressed and future work is required to determine if this method is sufficient for the labeling and enrichment of natively expressed plant proteins and if it is amenable to subsequent mass spectrometry analysis.

## Discussion

We describe the development of a click chemistry-based method using metabolic labeling with ω-alkynyl fatty acid analogs to study the fatty acylation, especially myristoylation, of both host and pathogen proteins in the plant cell. Our data directly demonstrate that the FLS2 receptor is S-acylated and the AvrPto effector that targets this sensor protein is subject to myristoylation and possibly S-acylation, supporting previous findings that strongly suggested these proteins were subject to such modifications in plants [20, 32]. Using our approach we also recapitulated an experiment demonstrating the myristoylation of Pto, the resistance protein responsible for recognition of AvrPto, that was initially performed with radiolabeled myristic acid and we were able to reduce the exposure time required to detect this fatty acid modification from a month to less than a minute [60]. Notably, these results were obtained using a combination of biotin and fluorescent reporters from an array of plant-based expression systems, including transiently transformed Arabidopsis protoplasts, stably transformed Arabidopsis plants, and transiently transformed *N. benthamiana* leaf tissue.

There are several examples demonstrating that feeding cultured cells fatty acid analogs results in their incorporation into cellular proteins through native metabolic mechanisms, without any obvious disruption to cellular processes [42, 44]. Metabolic labeling of proteins with ‘clickable’ fatty acid analogs has several advantages over the use of radiolabeled fatty acids, GC–MS approaches, and the ABE method for investigating protein fatty acylation. Proteins metabolically labeled with these bio-orthogonal analogs can be selectively and covalently modified with a variety of secondary tags via click chemistry. These secondary tags can include various affinity purification groups, such as biotin, or fluorescent reporter dyes. The click-mediated addition of affinity purification tags enables the capture and analysis of proteins modified by a given fatty acid and unlike the ABE and GC–MS approaches this technique can be applied to the study of myristoylated proteins. A further advantage over the ABE method is the decisive nature of the labeling offered with the fatty acid analogs. The direct tagging of proteins metabolically labeled with a given fatty acid analog, rather than the removal and replacement of all protein thioesters, can avoid much of the ambiguity and false positives associated with the ABE approach [35, 39–41].

It should be noted that the ABE method does not require feeding fatty acids to the cells or organism of interest, which is a disadvantage of metabolic labeling approaches using either radiolabeled or ω-alkynyl/-azido fatty acid analogs. However, these feeding approaches make it possible to perform pulse-chase experiments, enabling the study of S-acylation turnover dynamics which have been shown to regulate plant protein function [11, 41]. Therefore, the methods described here have the potential to enable dynamic protein S-acylation analysis in plants without the need for radioactive materials [40]. The incubation period is critical to the success of all labeling experiments and may have to be adjusted depending on the fatty acid analog, protein of interest, and metabolism of the system under investigation [40]. The major advantages of the clickable fatty acid analogs over radiolabeled fatty acids, beyond enabling proteome-wide enrichment of proteins modified by a particular form of fatty acylation, are the nonradioactive nature of these reagents and the signal strength of the click-compatible biotin and fluorescent reporters. A study comparing the detection of protein myristoylation using [^3^H]-myristic acid versus that produced by ω-azido myristic acid, with subsequent biotinylation, showed that the latter produced signal intensities up to one million times stronger than that of the tritiated fatty acid [84]. Similarly, using our technique we were able to detect myristoylation of Pto transiently expressed in *N. benthamiana* with exposure times of less than one minute, in contrast to the month-long exposure time required to see the signal for the fatty acylation of a similarly expressed and immunoprecipitated Pto with tritiated myristic acid [60].

Unlike myristoylation, S-acylation is a reversible modification mainly due to the thioester bond between the cysteine side chain and the fatty acid, which is less stable than the amide linkage responsible for coupling myristate to an N-terminal glycine [40, 85]. The strong amide attachment of the Alk12 myristic acid analog as a result of myristoylation-type modifications provides a stable handle for protein purification and detection, which has worked very reliably in our hands. In contrast, the labile nature of S-acylation requires more delicate handling, particularly during elution steps following enrichment via immunoprecipitation procedures [40, 85]. Whereas myristoylation labeling was resistant to 10 % 2-mercaptoethanol in the Laemmli sample buffer, in instances analyzing S-acylation the 2-mercaptoethanol concentrations were lowered to 0.1 % to preserve the thioester bonds [26, 40, 85]. We found that the use of fluorescent dyes is preferable for the study of S-acylation because it allows rapid in-gel detection and does not require blotting of the labeled proteins, a process that can lead to thioester hydrolysis and loss of the reporter molecule [40, 42]. Even so, biotin-based reporters remain an attractive option because western blotting is highly sensitive and the materials are more readily accessible compared to fluorescent dye reagents that are expensive and require somewhat specialized scanners for detection.

Metabolic labeling methods, such as the one we present here, are generally acknowledged to avoid the false positive problems inherent to ABE-type approaches; however, labeling also has the potential for false positives. The fatty acid analogs can be metabolized into the cellular lipid pools if the labeling period is too long, resulting in non-target fatty acids possessing the alkyne moiety which can yield false positives [40, 44]. For this reason it is imperative to determine the fatty acid analog incubation period for a given protein and/or plant system and note that this period might differ considerably from the times compatible with the proteins in our particular study [45, 50]. It has also been reported that Alk12 and Alk14 can participate in both myristoylation and S-acylation, which was attributed to a lack of specificity in the fatty acylation machinery rather than metabolism of the fatty acid analogs [42]. Alk16 on the other hand seems to more specifically label S-acylated proteins and might be a better choice for detecting this specific form of fatty acid modification [42, 86]. Another potential source for false positives when using labeling approaches is the addition of fatty acids to non-target amino acids. The most notable example of this phenomenon is the labeling of the G2A mutant of the mammalian membrane-associated non-tyrosine protein kinase Fyn, the native form of which is known to be subject to N-terminal dual fatty acylation, with both radioactive and alkyne bearing myristic acid analogs [42]. We also observed some instances of non-target labeling, primarily when working with the palmitic acid analog Alk14 and the stearic acid analog Alk16, which could be attributable to any of the phenomena described above (data not shown). It should be noted that the unique sensitivity of S-acyl adducts to treatments with strong reducing agents and nucleophiles such as 2-mercaptoethanol, dithiothreitol, and hydroxylamine can be leveraged to address some issues with ambiguous labeling [42]. In our experience, detection of myristoylation with the Alk12 reagent has been very specific because the G2A mutants reliably abolished labeling by the fatty acid analog. Another potential problem with labeling approaches is that the presumed overabundance of the fatty acid analogs in these feeding assays leads to unspecific incorporation at non-target residues. However, our results with FLS2 would suggest that this may not be an issue because the C830S,C831S mutant appeared to abolish incorporation of the palmitic acid analog Alk14 despite being properly localized at the PM [20]. Toxicity of these fatty acid analogs can be a concern and should be evaluated for the plant system under investigation. For example, it was found that analogs of lauric acid bearing a terminal alkyne similar to the alkyne fatty acid analogs used in our study inhibited a lauric acid ω-hydroxylase in microsome preparations from *Vicia sativa* [87]. In our experiments with Arabidopsis protoplasts we observed strong phytotoxicity with the azide fatty acid analog Az12, however, the alkyne fatty acid analog Alk12 showed no adverse effects and for this reason we decided to use the alkyne-functionalized analogs for our work. It should be noted that the reagents were prepared in accordance with their manufacturers’ instructions, which called for the use of different solvents. The Az12 was prepared as a 40 mM stock in dimethyl sulfoxide (DMSO) and the Alk12 as a 50 mM stock in ethanol. The DMSO used for the Az12 stock solution could contribute to the observed toxicity, but in all treatments the stock solution was diluted at least 1000-fold, meaning that the plant cells were maximally exposed to 0.1 % DMSO. While we believe that the Az12 is most likely responsible for the observed toxicity to the protoplasts, we cannot rule out contributions from the DMSO.

Finally, our experiments were performed with overexpressed proteins and it will likely be more difficult to detect fatty acylation of natively expressed proteins. To test candidate proteins, transient overexpression with a commercial epitope tag is ideal because this enables the use of point mutants and allows for easy purification and concentration of the proteins prior to performing click chemistry. In our experience, amino acid substitutions that prevent protein fatty acylation and enable mapping of the modification to specific residues can result in protein instability and it can be helpful to employ fusions with green fluorescent protein variants to stabilize problematic substitution mutant proteins. In instances where the study of natively expressed proteins is desirable specific antibodies can be used to purify and concentrate the protein of interest, providing optimal buffer conditions for an efficient click reaction.

## Conclusions

We described the development and application of a metabolic labeling approach coupled with click chemistry to quickly and easily determine fatty acylation, especially myristoylation, of candidate proteins in plant cells. Our method can reduce the time required to assess protein fatty acid modifications from months to less than a week and relies on neither radioactivity nor mass spectrometry. We demonstrated the ability of our approach to determine the fatty acylation status of three representative proteins involved in plant-pathogen interactions using a variety of expression systems. Although presently most effective for determining protein myristoylation, this technique promises to provide mechanistic details of the molecular tactics used at the host plasma membrane in the battle between plants and pathogens. In addition, we expect that with some modifications this approach will be broadly applicable for the study of protein fatty acylation in plants and will shed light on new mechanisms not only involving plant-pathogen interactions but the wider field of plant biology.

## Methods

### Plant material

Seeds of *Arabidopsis thaliana* accession Columbia (Col-0) or the derived transgenic line conditionally expressing *avrPto* [68] were suspended in 0.1 % agarose and cold-stratified for 3 days at 4 °C. The plants were grown in a controlled environment chamber with 8 h light and 16 h dark periods at 22 °C and 20 °C, respectively, with 60 % relative humidity for 6 weeks. *Nicotiana benthamiana* accession Nb-1 [88] was grown in a controlled environment chamber with a light/dark cycle of 16 h and 8 h, respectively, with 65 % relative humidity and temperatures of 24 °C during light and 22 °C during dark periods for 4–5 weeks.

### Cloning

To generate the Gateway entry clones, complete open reading frames (ORFs) without the stop codons were amplified with Phusion DNA polymerase (cat. no. F-530S, Thermo Scientific) from existing plasmids. The ORFs were blunt-end ligated into the SmaI (cat. no. R0141S, New England Biolabs) site of pJLSmart [89] or pJM51 [90] with T4 DNA ligase (cat. no. M0202S, New England Biolabs). Point mutations were introduced using complementary custom DNA oligonucleotides (Integrated DNA Technologies) following standard protocols (e.g. Stratagene QuikChange site-directed mutagenesis kit). Entry clones were recombined into destination vectors using the LR Clonase II enzyme mix (cat. no. 11791-020, Invitrogen) following the manufacturer’s protocol. Destination vectors used were HBT95 [66] for protoplast expression and the pGWB series [91] for Agrobacterium-mediated transformation. All constructs were control digested with BsrGI (cat. no. R0575S, New England Biolabs) and sequence-verified prior to use. Sequencing services were provided by the Biotechnology Resource Center at Cornell University. The pBTEX constructs have been described previously [92, 93]. All vectors and constructs are listed in Additional file 5: Table S1.

### Protoplast isolation and transformation

Arabidopsis protoplasts were prepared and transformed as previously described [94, 95]. Briefly, the epidermis on the abaxial side of fully expanded leaves was peeled off and the leaves floated in protoplast isolation medium. The protoplasts were collected, washed, and the cell density adjusted. Plasmid DNA was added and the protoplasts were transformed by PEG-calcium transfection. Protoplasts were washed again and resuspended in the presence of the palmitic acid analog Alk14 (cat. no. 13266, Cayman Chemical) at a final concentration of 10 μM. Cells were incubated for 6 h, collected, and stored at −80 °C until further processing. For the comparison between azide and alkyne fatty acid analogs, transformed protoplasts were resuspended in the presence of the myristic acid analog Az12 (cat. no. C10268, Invitrogen) or Alk12 (cat. no. 13267, Cayman Chemical) at the indicated final concentrations. All fatty acid analogs were prepared following their manufacturers’ protocols and their structures a shown in Additional file 4: Figure S4. Cells were incubated overnight, collected, and protein levels analyzed by western blotting.

### Conditional expression of *avrPto* in transgenic Arabidopsis

Transgenic Arabidopsis conditionally expressing *avrPto* under control of a dexamethasone-inducible promoter [68] were sprayed with 20 μM dexamethasone (cat. no. D1756, Sigma-Aldrich) in 0.1 % ethanol with 0.01 % Silwet L-77 (cat. no. VIS-01, Lehle Seeds) to induce gene expression. Leaves were infiltrated twice with 10 μM myristic acid analog Alk12, 6 h after induction and 6 h before sampling. Tissue was collected 30 h after induction and stored at −80 °C until further processing.

### Agrobacterium-mediated transient expression

*Agrobacterium tumefaciens* strain GV3101 with helper plasmid pMP90 [96] was transformed with the pGWB constructs; the pBTEX constructs had previously been moved into *A. tumefaciens* strain GV2260 [93]. Confirmed strains were grown on lysogeny broth (LB) plates with appropriate antibiotics at 30 °C for 36–48 h. Bacteria were then scraped from plates and resuspended in infiltration buffer containing 10 mM MgCl_2_, 10 mM MES pH 5.7, and 200 μM acetosyringone (cat. no. D134406, Sigma-Aldrich). The OD_600_ was adjusted to a final density of 0.3 for each strain and the bacteria incubated for at least 1 h at room temperature. Leaves of *N. benthamiana* were infiltrated with needleless syringes and the plants placed on a shaded growth chamber shelf. Leaves were infiltrated twice with 10 μM Alk12, 24 h after Agrobacterium infiltration and 6 h before sampling. Tissue samples were collected 48 h after transformation. For the cell death assay, leave tissue was infiltrated once with 50 μM Alk12, Alk14, Alk16 (cat. no. 90270, Cayman Chemical), or buffer 24 h after Agrobacterium infiltration.

### Click reaction

Leaf tissue was ground with a TissueLyser II (Qiagen) and proteins extracted in ‘RIPA’ buffer containing 1 × PBS pH 7.4, 1 % v/v Triton X-100 (cat. no. X100, Sigma-Aldrich), 0.5 % w/v sodium deoxycholate, and 0.1 % w/v SDS, with EDTA-free protease inhibitor (cat. no. 05892791001, Roche Diagnostics). Protoplasts were lysed by brief vortexing in RIPA buffer with EDTA-free protease inhibitor. Affinity resin was added to the cleared supernatant and immunoprecipitation performed. FLS2-HA was purified with anti-HA (cat. no. E6779, Sigma-Aldrich); YFP fusions were purified with anti-GFP (cat. no. gta-10, ChromoTek); and untagged AvrPto was purified with custom anti-AvrPto antibody [32] coupled to protein A resin (cat. no. P6486, Sigma-Aldrich). Agarose beads were resuspended in RIPA buffer and the following components added for the click reaction: 500 μM BTTP ligand, 250 μM CuSO_4_, 2 mM sodium ascorbate, and 100 μM azide tag. The reaction was incubated for 1 h at room temperature or overnight at 4 °C and the beads were washed and resuspended in Laemmli sample buffer for protein detection. A detailed protocol is provided in Additional file 6: Methods S1. For the myristoylome pilot experiment, total protein was extracted in 1× PBS pH 7.4 with 4 % w/v SDS and EDTA-free protease inhibitor. Standard methanol/chloroform precipitation was used to purify and concentrate the proteins [97]. Click chemistry with the crude protein extract was performed in 1× PBS pH 7.4 with 4 % w/v SDS in the presence of 1 mM CuSO_4_ to attach a biotin tag to AvrPto. We found that higher concentrations of the copper catalyst are required for efficient click reactions in crude protein extracts. A second methanol/chloroform precipitation was used to clean the sample, proteins were resuspended in 1× PBS pH 7.4 with 4 % w/v SDS and EDTA-free protease inhibitor, diluted with 1× PBS pH 7.4 to reduce SDS concentration to around 0.7 %, and the myristoylated proteins enriched using streptavidin resin (cat. no. 20349, Thermo Scientific). The BTTP ligand 3-[4-({bis[(1-tert-butyl-1H-1,2,3-triazol-4-yl)methyl]amino}methyl)-1H-1,2,3-triazol-1-yl]propanol was a gift from Dr. Frank C. Schroeder (Boyce Thompson Institute for Plant Research and Department of Chemistry and Chemical Biology, Cornell University) and its structure is shown in Additional file 4: Figure S4. BTTP is not commercially available at the time of writing, but it can be obtained from the Chemical Biology Core Facility of the Albert Einstein College of Medicine (www.einstein.yu.edu/research/shared-facilities/chemical-biology/Ligands-for-CuAAC). The N_3_-biotin reagent biotin-PEG3-azide, used as both a reporter and affinity purification handle, was purchased from Click Chemistry Tools (cat. no. AZ104-10). The infrared fluorescent reporter IRDye 800CW azide was obtained from LI-COR Biosciences (cat. no. 929-60000).

### Protein detection

All samples were brought up in Laemmli sample buffer, with Orange G (cat. no. O3756, Sigma-Aldrich) substituted for bromophenol blue for the fluorescence imaging experiments to minimize signal interference. Gel electrophoresis and western blotting was performed following standard protocols (e.g. Bio-Rad bulletin 6040 and 2895, respectively). Detection of attached infrared fluorescent dye was performed using an Odyssey infrared imager (LI-COR Biosciences) after fixing the gel by incubation in 40 % methanol and 10 % acetic acid protected from light with gentle shaking overnight at room temperature. In some instances multiple incubations in fixing buffer were required to remove background signal. Attached biotin was detected using streptavidin-HRP (cat. no. S-911, Invitrogen); untagged AvrPto was detected using custom anti-AvrPto antibody followed by anti-rabbit-HRP (cat. no. W4011, Promega); FLS2-HA and AvrPto-HA were detected using anti-HA-HRP (cat. no. 12013819001, Roche Diagnostics); YFP fusion proteins were detected using anti-GFP (cat. no. 11814460001, Roche Diagnostics) followed by anti-mouse-HRP (cat. no. sc-2005, Santa Cruz Biotechnology); and YFP-FLAG was detected with anti-FLAG-HRP (cat. no. A8592, Sigma-Aldrich).

## Additional files

10.1186/s13007-016-0138-2 Azide fatty acid analogs, but not alkyne fatty acid analogs, interfere with cellular functions. **(A)** Arabidopsis protoplasts were transformed with FLAG epitope-tagged *YFP* and treated with different concentrations of the azide fatty acid analog Az12. Cells were incubated overnight and total protein extracted. Anti-FLAG western blotting was used to detect YFP accumulation. Coomassie brilliant blue (CBB) stain was used to visualize total protein and demonstrate equal loading. NT, not transformed. Black dividing lines indicate removal of irrelevant lanes from the blot and gel images. **(B)** Arabidopsis protoplasts were transformed with FLAG epitope-tagged *YFP* and treated with different concentrations of the alkyne fatty acid analog Alk12. Cells were incubated overnight and total protein extracted. Anti-FLAG western blotting was used to detect YFP accumulation. CBB stain was used to visualize total protein and demonstrate equal loading.
10.1186/s13007-016-0138-2 Alkyne fatty acid analogs do not interfere with immunity mechanisms and are incorporated in fatty acylated proteins in the context of intact plant leaf tissue. **(A)** *Nicotiana benthamiana* leaves were infiltrated with Agrobacterium strains carrying *Pto* and empty vector (EV) or *avrPto*. 50 μM Alk12, Alk14, Alk16, or buffer were infiltrated 24 h after Agrobacterium infiltration. Plants were monitored for programmed cell death and pictures taken 2 days after transformation. **(B)** *Nicotiana benthamiana* was used to transiently express HA epitope-tagged *avrPto*. 10 μM Alk12, Alk14, Alk16, or buffer was infiltrated twice, 24 h after Agrobacterium infiltration and 6 h before sampling. Tissue was collected 48 h after transformation, total protein extracted, AvrPto immunoprecipitated using anti-HA resin, and a fluorescent tag added using click chemistry. Incorporated alkyne fatty acid analogs were visualized by fluorescence imaging and total protein was detected by anti-HA western blotting. Black dividing lines indicate removal of irrelevant lanes from the blot and gel images.
10.1186/s13007-016-0138-2 Protein capture by means of myristoylation provides a potential method for the enrichment and investigation of the plant myristoylome. **(A)** Modified experimental scheme to capture and enrich myristoylated proteins using AvrPto as a test protein. **(B)** *Nicotiana benthamiana* was used to transiently express HA epitope-tagged *avrPto*. 50 μM Alk12 was infiltrated twice, 24 h after Agrobacterium infiltration and 6 h before sampling. Tissue was collected 48 h after transformation, total protein extracted, and a biotin tag added using click chemistry. Streptavidin affinity purification was used to enrich biotinylated proteins and AvrPto was detected using anti-HA western blotting. Input shows AvrPto levels before affinity purification.
10.1186/s13007-016-0138-2 Structures of the fatty acid analogs and ligands used in this study. **(A)** Myristic acid analog Alk12. **(B)** Palmitic acid analog Alk14. **(C)** Stearic acid analog Alk16. **(D)** BTTP ligand 3-[4-({bis[(1-tert-butyl-1H-1,2,3-triazol-4-yl)methyl]amino}methyl)-1H-1,2,3-triazol-1-yl]propanol.
10.1186/s13007-016-0138-2 Vectors and plasmids used in this study.
10.1186/s13007-016-0138-2 Detailed click reaction protocol.
