# Supplementary figures and images for: Detecting N-myristoylation and S-acylation of host and pathogen proteins in plants using click chemistry
Source: Plant Methods. 2016 Aug 3;12:38. doi: 10.1186/s13007-016-0138-2 (PMC4972946; doi:10.1186/s13007-016-0138-2)

Figure S1

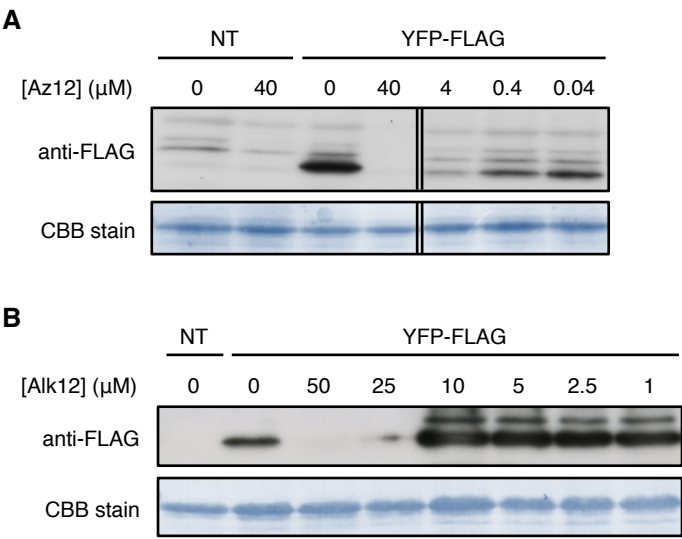

Supplement: Supplementary file 1 — 10.1186/s13007-016-0138-2 Azide fatty acid analogs, but not alkyne fatty acid analogs, interfere with cellular functions. (A) Arabidopsis protoplasts were transformed with FLAG epitope-tagged YFP and treated with different concentrations of the azide fatty acid analog Az12. Cells were incubated overnight and total protein extracted. Anti-FLAG western blotting was used to detect YFP accumulation. Coomassie brilliant blue (CBB) stain was used to visualize total protein and demonstrate equal loading. NT, not transformed. Black dividing lines indicate removal of irrelevant lanes from the blot and gel images. (B) Arabidopsis protoplasts were transformed with FLAG epitope-tagged YFP and treated with different concentrations of the alkyne fatty acid analog Alk12. Cells were incubated overnight and total protein extracted. Anti-FLAG western blotting was used to detect YFP accumulation. CBB stain was used to visualize total protein and demonstrate equal loading. [file 13007_2016_138_MOESM1_ESM.pdf]

Figure S2

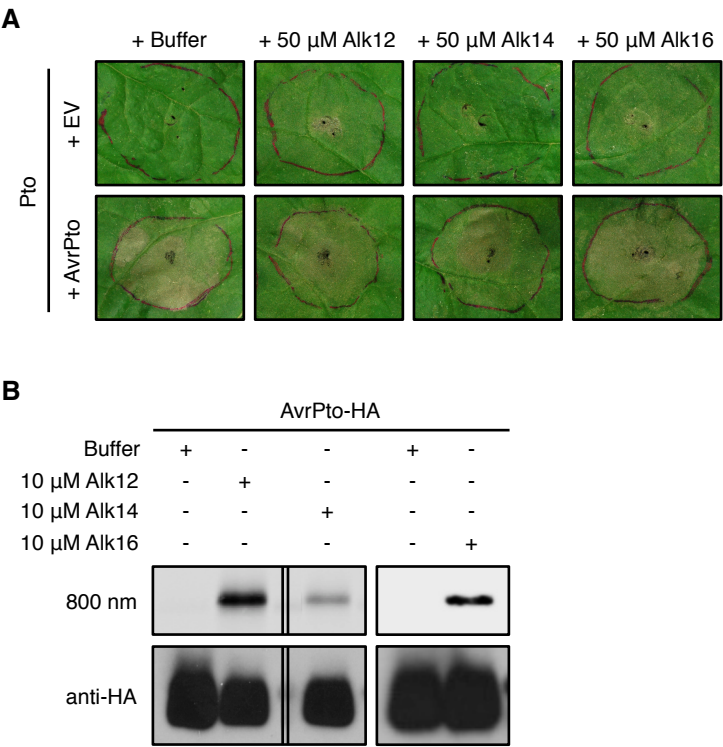

Supplement: Supplementary file 2 — 10.1186/s13007-016-0138-2 Alkyne fatty acid analogs do not interfere with immunity mechanisms and are incorporated in fatty acylated proteins in the context of intact plant leaf tissue. (A) Nicotiana benthamiana leaves were infiltrated with Agrobacterium strains carrying Pto and empty vector (EV) or avrPto. 50 μM Alk12, Alk14, Alk16, or buffer were infiltrated 24 h after Agrobacterium infiltration. Plants were monitored for programmed cell death and pictures taken 2 days after transformation. (B) Nicotiana benthamiana was used to transiently express HA epitope-tagged avrPto. 10 μM Alk12, Alk14, Alk16, or buffer was infiltrated twice, 24 h after Agrobacterium infiltration and 6 h before sampling. Tissue was collected 48 h after transformation, total protein extracted, AvrPto immunoprecipitated using anti-HA resin, and a fluorescent tag added using click chemistry. Incorporated alkyne fatty acid analogs were visualized by fluorescence imaging and total protein was detected by anti-HA western blotting. Black dividing lines indicate removal of irrelevant lanes from the blot and gel images. [file 13007_2016_138_MOESM2_ESM.pdf]

Figure S4

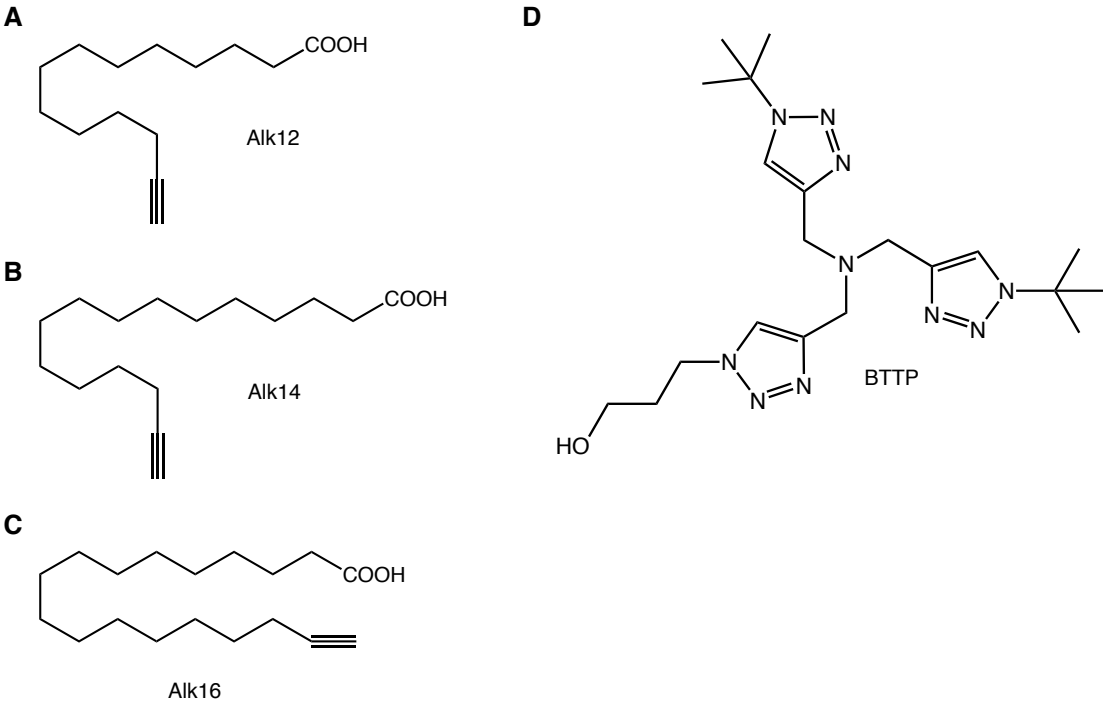

Supplement: Supplementary file 4 — 10.1186/s13007-016-0138-2 Structures of the fatty acid analogs and ligands used in this study. (A) Myristic acid analog Alk12. (B) Palmitic acid analog Alk14. (C) Stearic acid analog Alk16. (D) BTTP ligand 3-[4-({bis[(1-tert-butyl-1H-1,2,3-triazol-4-yl)methyl]amino}methyl)-1H-1,2,3-triazol-1-yl]propanol. [file 13007_2016_138_MOESM4_ESM.pdf]
