# Supplementary material for: Detecting N-myristoylation and S-acylation of host and pathogen proteins in plants using click chemistry
Source: Plant Methods. 2016 Aug 3;12:38. doi: 10.1186/s13007-016-0138-2 (PMC4972946; doi:10.1186/s13007-016-0138-2)
Supplement: Supplementary file 3 — 10.1186/s13007-016-0138-2 Protein capture by means of myristoylation provides a potential method for the enrichment and investigation of the plant myristoylome. (A) Modified experimental scheme to capture and enrich myristoylated proteins using AvrPto as a test protein. (B) Nicotiana benthamiana was used to transiently express HA epitope-tagged avrPto. 50 μM Alk12 was infiltrated twice, 24 h after Agrobacterium infiltration and 6 h before sampling. Tissue was collected 48 h after transformation, total protein extracted, and a biotin tag added using click chemistry. Streptavidin affinity purification was used to enrich biotinylated proteins and AvrPto was detected using anti-HA western blotting. Input shows AvrPto levels before affinity purification. [file 13007_2016_138_MOESM3_ESM.pdf]

Figure S3

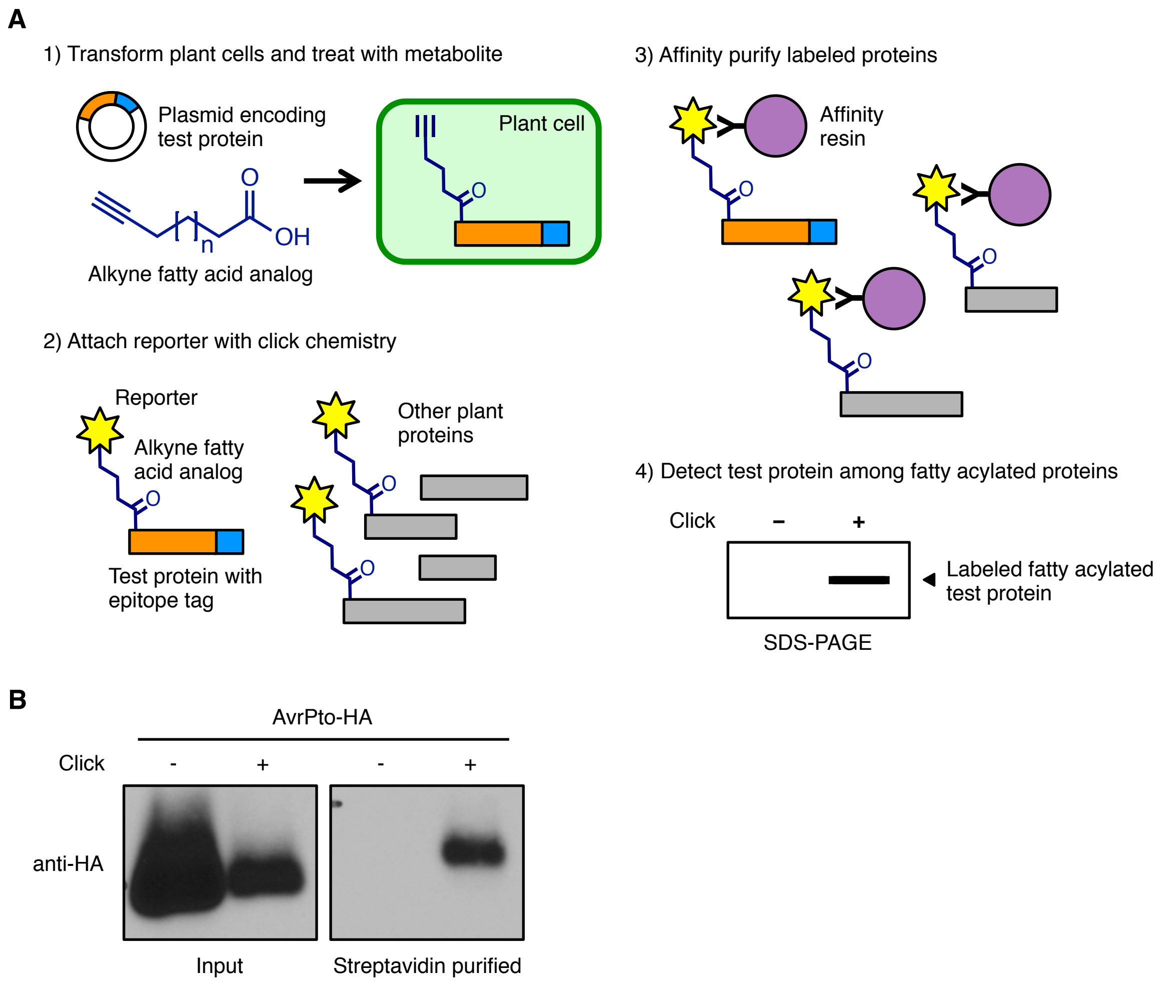
A
1) Transform plant cells and treat with metabolite
Plasmid encoding test protein
O
OH
n
Alkyne fatty acid analog
Plant cell
O
2) Attach reporter with click chemistry
Reporter
Alkyne fatty acid analog
O
Test protein with epitope tag
Other plant proteins
O
O
3) Affinity purify labeled proteins
Affinity resin
O
O
O
4) Detect test protein among fatty acylated proteins
Click
–
+
Labeled fatty acylated test protein
SDS-PAGE
B
AvrPto-HA
Click
-
+
-
+
anti-HA
Input
Streptavidin purified
