# Supplementary material for: Detecting N-myristoylation and S-acylation of host and pathogen proteins in plants using click chemistry
Source: Plant Methods. 2016 Aug 3;12:38. doi: 10.1186/s13007-016-0138-2 (PMC4972946; doi:10.1186/s13007-016-0138-2)
Supplement: Supplementary file 5 — 10.1186/s13007-016-0138-2 Vectors and plasmids used in this study. [file 13007_2016_138_MOESM5_ESM.pdf]

Table S1. Vectors and plasmids used in this study.

Vector	Insert	Identifier	Purpose/description	Source
pJLSmart	-	-	Gateway entry vector	[89]
pJLSmart	AvrPto [no stop]	pPB2	For LR recombination into destination vectors	This work
pJLSmart	AvrPto (G2A) [no stop]	pPB105	For LR recombination into destination vectors	This work
pJLSmart	FLS2 [no stop]	pJM379	For LR recombination into destination vectors	This work
pJLSmart	FLS2 (C830S,C831S) [no stop]	pPB153	For LR recombination into destination vectors	This work
pJM51	-	-	Modified pJLSmart Gateway entry vector with Shine-Dalgarno sequence	[90]
pJM51	YFP [no stop]	pSS22	For LR recombination into destination vectors	This work
HBT95-HA	-	-	Gateway destination vector for protoplast expression with C-terminal 2x HA tag	[66]
HBT95-HA	FLS2	pSS122	Generating C-terminal HA fusion	This work
HBT95-HA	FLS2 (C830S,C831S)	pPB154	Generating C-terminal HA fusion	This work
HBT95-FLAG	-	-	Gateway destination vector for protoplast expression with C-terminal FLAG tag	[66]
HBT95-FLAG	YFP	pSS298	Generating C-terminal FLAG fusion	This work
pGWB411	-	-	Binary gateway destination vector for plant transformation with C-terminal FLAG tag	[91]
pGWB411	YFP	pSS190	Generating C-terminal FLAG fusion	This work
pGWB541	-	-	Binary gateway destination vector for plant transformation with C-terminal eYFP tag	[91]
pGWB541	AvrPto	pSS349	Generating C-terminal eYFP fusion	This work
pGWB541	AvrPto (G2A)	pSS350	Generating C-terminal eYFP fusion	This work
pBTEX	-	-	Binary expression vector for plant transformation	[92]
pBTEX	AvrPto-HA	-	Binary expression vector for plant transformation	[93]
pBTEX	Pto-HA	-	Binary expression vector for plant transformation	[93]
pBTEX	Pto-YFP	-	Binary expression vector for plant transformation	This work
pBTEX	Pto (G2A)-YFP	-	Binary expression vector for plant transformation	This work

66. He P, Shan L, Lin N-C, Martin GB, Kemmerling B, Nürnberger T, et al. Specific bacterial suppressors of MAMP signaling upstream of MAPKKK in *Arabidopsis* innate immunity. Cell. 2006;125:563–75.

89. Mathieu J, Warthmann N, Küttner F, Schmid M. Export of FT protein from phloem companion cells is sufficient for floral induction in *Arabidopsis.* Curr Biol. 2007;17:1055–60.

90. Cheng W, Munkvold KR, Gao H, Mathieu J, Schwizer S, Wang S, et al. Structural analysis of *Pseudomonas syringae* AvrPtoB bound to host BAK1 reveals two similar kinase-interacting domains in a type III effector. Cell Host Microbe. 2011;10:616–26.

91. Nakagawa T, Suzuki T, Murata S, Nakamura S, Hino T, Maeo K, et al. Improved Gateway binary vectors: high-performance vectors for creation of fusion constructs in transgenic analysis of plants. Biosci Biotechnol Biochem. 2007;71:2095–100.

92. Frederick RD, Thilmony RL, Sessa G, Martin GB. Recognition specificity for the bacterial avirulence protein AvrPto is determined by Thr-204 in the activation loop of the tomato Pto kinase. Mol Cell. 1998;2:241–5.

93. Abramovitch RB, Kim Y-J, Chen S, Dickman MB, Martin GB. *Pseudomonas* type III effector AvrPtoB induces plant disease susceptibility by inhibition of host programmed cell death. EMBO J. 2003;22:60–9.
