# Supplementary material for: Detecting N-myristoylation and S-acylation of host and pathogen proteins in plants using click chemistry
Source: Plant Methods. 2016 Aug 3;12:38. doi: 10.1186/s13007-016-0138-2 (PMC4972946; doi:10.1186/s13007-016-0138-2)
Supplement: Supplementary file 6 — 10.1186/s13007-016-0138-2 Detailed click reaction protocol. [file 13007_2016_138_MOESM6_ESM.pdf]

Methods S1. Detailed experimental protocol.

A) Fatty acid analog preparation and infiltration

Timing of fatty acid analog infiltration for transiently expressed genes in Nicotiana benthamiana assuming that maximum protein accumulation occurs after approximately 48 h.

1) Prepare each alkyne fatty acid analog (Alk12, Alk14, Alk16) as a 50 mM stock in absolute ethanol and store at -20ºC.
2) Immediately before use, dilute the fatty acid analog stock in water to 10-50 µM. Note that the stock preparations might require brief water bath sonication and/or vortexing upon thawing to ensure resuspension of the analog.
3) Infiltrate leaves with 10-50 µM alkyne fatty acid analog using a blunt syringe 24 h after agroinfiltration.
4) Repeat the fatty acid analog infiltration 6 h before sampling (i.e., 42 h after agroinfiltration).

B) Protein extraction

1) Collect leaf samples expressing the protein of interest (about 0.1 g for *N. benthamiana* leaves and depending on protein expression levels).
2) Grind leaf samples to a fine powder in liquid nitrogen.
3) Remove steel beads if used and add 1 ml cold RIPA buffer (1x PBS pH 7.4, 1% v/v Triton X-100, 0.5% w/v sodium deoxycholate, 0.1% w/v SDS) with EDTA-free protease inhibitor cocktail (e.g. Roche cOmplete ULTRA mini EDTA-free; RIPA-X).
4) Incubate on rotator for 15 min at 4ºC.
5) Centrifuge at 16,000 x *g* for 5 min at 4ºC and transfer supernatant to fresh 1.5 ml tube.
6) Repeat step 5) to remove leftover debris.

C) Affinity purification

1) Wash affinity resin twice with 1 ml cold RIPA buffer (use about 20 µl resin suspension per sample; centrifuge at 0.5 x *g* for 30 s for all resin wash steps).
2) Resuspend resin in cold RIPA buffer to the original volume.
3) Add 20 µl washed resin suspension to each sample supernatant.
4) Incubate on rotator for about 4 h at 4ºC.
5) Carefully remove supernatant and wash resin twice with 1 ml cold 1x PBS pH 7.4.
6) Transfer resin to 600 µl tube and wash three times with 500 µl cold RIPA buffer.

D) Click reaction

1) Resuspend resin in 93 µl cold RIPA or RIPA-X buffer.
2) Add 7 µl click master mix (see section **E**).
3) Incubate on rotator 1 h at room temperature or overnight at 4ºC.
4) Wash resin twice with 500 µl cold 1x PBS pH 7.4 and three times with 500 µl cold RIPA buffer.
5) Resuspend resin in 20 µl 2x Laemmli sample buffer with 10% 2-mercaptoethanol (use 0.1% for S-acylation experiments to prevent reduction of the thioester bonds).
6) Boil samples for 5-10 min and use for gel electrophoresis or store at -20ºC.

E) Click master mix and ligand preparation

2 µl BTTP ligand (25 mM stock; see below)
1 µl $CuSO_4$ (25 mM stock; dissolved in water)
2 µl Sodium ascorbate (100 mM stock; dissolved in water)
2 µl Azide tag (5 mM stock; dissolved in water; stored at -20ºC)
7 µl

Prepare the BTTP ligand as a 25 mM stock by initially dissolving the material in 1 part absolute ethanol by water bath sonication and/or vortexing and then diluting to the final volume using 4 parts water. The final solution will have a pale yellow/brown color and should be stored at -20ºC. Note that vortexing might be required to fully resuspend the ligand after thawing.

F) Additional notes

- All buffers and reagents are prepared at room temperature unless otherwise noted.
- Protease inhibitor cocktail should be added the day of the experiment.
- $CuSO_4$ and sodium ascorbate solutions should be prepared fresh.
- Click reactions using the fluorescent dye should be incubated in the dark.
- If no substitution mutant is available, $CuSO_4$ can be replaced with water as a negative control.
